# Targeted deep sequencing of flowering regulators in *Brassica napus* reveals extensive copy number variation

**DOI:** 10.1038/sdata.2017.13

**Published:** 2017-03-14

**Authors:** Sarah Schiessl, Bruno Huettel, Diana Kuehn, Richard Reinhardt, Rod J. Snowdon

**Affiliations:** 1 Department of Plant Breeding, Justus Liebig University, IFZ Research Centre for Biosystems, Land Use and Nutrition, Heinrich-Buff-Ring 26-32, 35392 Giessen, Germany; 2 Max Planck Institute for Breeding Research, Carl-von-Linné-Weg 10, 50829 Cologne, Germany

**Keywords:** Agricultural genetics, Evolutionary biology, DNA sequencing, Polyploidy in plants, Plant breeding

## Abstract

Gene copy number variation (CNV) is increasingly implicated in control of complex trait networks, particularly in polyploid plants like rapeseed (*Brassica napus* L.) with an evolutionary history of genome restructuring. Here we performed sequence capture to assay nucleotide variation and CNV in a panel of central flowering time regulatory genes across a species-wide diversity set of 280 *B. napus* accessions. The genes were chosen based on prior knowledge from *Arabidopsis thaliana* and related *Brassica* species. Target enrichment was performed using the Agilent SureSelect technology, followed by Illumina sequencing. A bait (probe) pool was developed based on results of a preliminary experiment with representatives from different *B. napus* morphotypes. A very high mean target coverage of ~670x allowed reliable calling of CNV, single nucleotide polymorphisms (SNPs) and insertion-deletion (InDel) polymorphisms. No accession exhibited no CNV, and at least one homolog of every gene we investigated showed CNV in some accessions. Some CNV appear more often in specific morphotypes, indicating a role in diversification.

## Background & Summary

Polyploid genomes present major challenges for DNA sequence analysis due to their high redundancy. Moreover, inter-subgenomic homology is a driving force for genomic rearrangements^1–3^, including translocations, inversions, duplications, deletions or homeologous non-reciprocal translocations (HNRTs)^1^. Translocations are events where genomic blocks are transferred to another chromosomal location in the same orientation, whereas inversions switch the orientation. Duplications are events where genetic regions are copied to another locus, meaning that the affected region increases in copy number, whereas deletions involve a loss of genetic regions from the genome and therefore decrease the copy number of the genes therein. HNRTs are coupled duplication-deletion events, where one region of the genome replaces a respective homeologous genome region. Changes in the frequency of genes or other genomic loci due to duplications, deletions and HNRTs are collectively described as copy number variation (CNV).

*Brassica napus* (oilseed rape, canola) has become a model plant for studies of polyploidy effects^4^, having been studied for genomic rearrangements over decades^5,6^. The strongest effects of genomic rearrangements, with regard to both size and abundance, were observed in synthetic, or resynthesized *B. napus*^1,2,7,8^. This indicates that substantial genomic rearrangements occur in the first generations after polyploidisation and are thereafter subjected to selection and fixation^2^. All the same, CNV can arise at any time and is therefore frequently observed^3^. With increasing reports associating CNV to major traits in different crop species, like wheat, maize and potato^9–11^, their detection is growing in importance for genomics-assisted breeding approaches to improve crop adaptation and yield.

Flowering time is an extremely important adaptive trait, with wide-ranging implications for the breeding process in crops like *B. napus* that are grown in different ecogeographical regions. Flowering time determines the lifecycle to be either annual or biannual and is also important for local adaptation. Biannual and annual *B. napus* forms have divergent lifecycles, and hence must be bred in separate breeding programs, which in turn lowers diversity in these two genetic pools. While extremely important, flowering time regulation is also highly complex, with studies in model species^12,13^ implicating over 200 different genes and non-coding RNAs. Polyploidy complicates this network not only by multiplying the gene copy number, but also by invoking potential for gene subfunctionalisation^14^. Knowledge of the underlying natural variation among *B. napus* flowering time regulators can help to understand which gene copies are decisive for restoring life cycle traits after introgression from a different gene pool. CNVs can play a central role in such variation^15^.

Different methodologies have been proposed for the detection of CNVs, either based on hybridization arrays or on next-generation sequencing (NGS) technologies^3^. As the latter deliver additional information like SNPs and InDels, they are better suited to unravel causal variants for trait variation^16^. Although different approaches exist to calculate CNVs from NGS data, the simplest and most abundant approach is the approximation by calculation read depth or sequence coverage. The read depth approach is based on the assumption that sequencing reads distribute equally over the sequenced space, so that deviations in coverage can be interpreted to indicate deletions or duplications. However, for cost-effective application of DNA resequencing on large panels of genotypes, the sequencing power should be focused on regions of interest, like exomes or groups of genes. This can be done by prior enrichment for loci of interest by hybridization with probes or baits, and different commercial solutions are available for this^17^. Here, we used the Agilent SureSelect technology (Agilent Inc., Santa Clara, CA, USA) for target enrichment. The resulting sequence capture libraries were subjected to Illumina sequencing in single-end mode. Baits were designed for homologs of 35 *Arabidopsis thaliana* flowering genes, based on sequences from *B. napus* along with its diploid progenitors *B. rapa and B. oleracea*, and used for production of 120mer RNA oligonucleotides for hybridization. Sequencing was performed for 280 diverse inbred lines as a part of the ERANET-ASSYST *B. napus* diversity panel^18^, containing annual and biannual oilseed, fodder, vegetable and rutabaga forms, along with exotic accessions. The results show a vast variance of SNPs, InDels and CNVs in the different breeding pools. An accompanying publication describes the biological implications of the data^15^.

## Methods

### Plant Material and phenotyping

A panel of 280 genetically diverse *B. napus* inbred lines (self-pollinated for 5 or more generations) was grown in in Giessen, Germany (50° 35′ N, 8° 40′ E) in 2012. The plant material was part of the ERANET-ASSYST *B. napus* diversity set, described in detail by^18–20^. Biannuals were grown in autumn-sown trials, whereas annual accessions were grown in spring-sown trials. Plots were sown in a completely randomized block design with a harvest plot size of 3×1.25 m in 1 replicate (containing around 200 plants).

### DNA isolation

Leaf material for genomic DNA extraction was harvested in spring 2012 from the field trial in Giessen, Germany. Pooled leaf samples were taken from at least 5 different plants per genotype, immediately shock-frozen in liquid nitrogen and kept at −20 °C until extraction. Leaf material was ground in liquid nitrogen with a mortar and pestle. DNA was extracted using a common CTAB protocol modified from Doyle and Doyle (1990) as described earlier^16^. DNA concentration was determined using a Qubit fluorometer and the Qubit dsDNA BR assay kit (Life Technologies, Darmstadt, Germany) according to the manufacturer’s protocol. DNA quantity and purity was further checked on 0.5% agarose gel (3 V/cm, 0.5xTBE, 120 min).

### Selection of target genes

As described in ref. 16, a set of 29 *A. thaliana* flowering time genes was selected to cover the entire genetic network controlling flowering time, including circadian clock regulators *(CYCLING DOF FACTOR 1 (CDF1), EARLY FLOWERING 3 (ELF3), GIGANTEA (GI)* and *ZEITLUPE (ZTL)*), the input pathways for vernalisation (*EARLY FLOWERING 7 (ELF7), EARLY FLOWERING IN SHORT DAYS (EFS), FLOWERING LOCUS C (FLC), FRIGIDA (FRI), SHORT VEGTATIVE PHASE (SVP), SUPPRESSOR OF FRIGIDA 4 (SUF4), TERMINAL FLOWER 2 (TFL2), VERNALISATION 2 (VRN2), VERNALISATION INSENSITIVE 3 (VIN3)),* photoperiod sensitivity (*CONSTANS (CO), CRYPTOCHROME 2 (CRY2), PHYTOCHROME A (PHYA), PHYTOCHROME B (PHYB))* and gibberellin (*GIBBERELLIN-3-OXIDASE 1 (GA3ox1)*), along with downstream signal transducers (*AGAMOUS-LIKE 24 (AGL24), APETALA 1 (AP1), CAULIFLOWER (CAL), FLOWERING LOCUS D (FD), FLOWERING LOCUS T (FT), FRUITFUL (FUL), LEAFY (LFY), SQUAMOSA PROMOTOR PROTEIN LIKE 3 (SPL3), SUPPRESSOR OF CONSTANS 1 (SOC1), TEMPRANILLO 1 (TEM1), TERMINAL FLOWER 1 (TFL1))*. On top, we also included 6 further genes: *CIRCADIAN CLOCK ASSISTED 1 (CCA1), FLAGELLIN-SENSITIVE 2 (FLS2), GLYCIN-RICH PROTEIN 7 (GRP7), GLYCIN-RICH PROTEIN 8 (GRP8), GORDITA (GORD)* and *SENSITIVITY TO RED LIGHT REDUCED 1 (SRR1),* giving a total of 35 genes. The respective Arabidopsis identifiers are given in Supplementary Table S1.

### Bait development

Before resequencing the total set of 280 accessions, 4 accessions representing divergent morphotypes were resequenced in a preliminary experiment in order to refine the bait pool (described in ref. 16). At the time, no reference sequence for *B. napus* was available. Baits were therefore produced on sequences of *B. rapa* and *B. oleracea*, using the program eArrayXD. The only exception was the target gene *FT*, for which promotor and gene sequences from *B. napus* were kindly made available by Carlos Molina, Christian Albrecht University of Kiel, Germany. A pre-publication draft (version 4.0) of the *B. napus* ‘Darmor-Bzh’ reference genome sequence assembly became available prior to public release, by generosity of Boulos Chalhoub, INRA, France, Unité de Recherche en Génomique Végétale.

Based on the mapping results of the preliminary experiment, the bait pool was modified in order to improve specificity. Enriched regions found in ref. 16 were classified into target regions and non-target regions by BLAST against the nucleotide database in NCBI. The bait pool was blasted against target and non-target regions (E-value cut-off e^−10^). Baits which had excessive non-target hits were manually removed. This was the case for bait groups on the target genes *FT, FUL* and *PHYA*. For some bait groups (*AP1, CO, SOC1*), too many baits (>30%) were deleted. In these cases, baits groups (120mer oligonucleotide sequences) were created using the *B. napus* pre-publication draft reference genome sequence assembly, with the Agilent Genomic Workbench program SureDesign (Agilent Inc., Santa Clara, CA, USA). These replaced the corresponding bait groups developed previously using *B. rapa* or *B. oleracea*. Bait groups were created using the ‘Bait Tiling’ tool. The parameters were set as follows: Sequencing Technology: ‘Illumina’, Sequencing Protocol: ‘Paired-End long Read (75 bp+)’, ‘Use Optimized Parameters (Bait length 120, Tiling Frequency 1x)’, Avoid Overlap: ‘20’, ‘User defined genome’, ‘Avoid Standard Repeat Masked Regions’. Baits for genes on the minus-strand were developed in sense, while baits on the plus-strand were developed in antisense.

In total, 63 bait groups were created for *B. rapa* homologs of the target genes, 71 bait groups for *B. oleracea* homologs and 24 bait groups for *B. napus* homologs.

### Sequence capture and sequencing

Custom bait production was carried out by Agilent Technologies (Agilent Inc., Santa Clara, CA, USA) using the output oligonucleotide sequences from SureDesign. Sequence capture was performed using the SureSelectXT 1 kb–499 kb Custom Kit (Agilent Inc., Santa Clara, CA, USA) according to the manufacturer’s instructions. The resulting TruSeq DNA library (Illumina Inc., San Diego, CA, USA) was sequenced on an Illumina HiSeq 2500 sequencer at the Max Planck Institute for Breeding Research (Cologne, Germany) in 100 bp single read mode.

### Sequence data analysis

#### Mapping

Quality control of the raw sequencing data was performed using FASTQC. Reads were mapped onto version 4.1 of the *B. napus* ‘Darmor-Bzh’ reference genome sequence assembly^21^. Mapping was performed using the software SOAP2 (http://soap.genomics.org.cn/soapaligner.html), with default settings and alternatively using the option r=0 to extract uniquely aligned reads. Removal of duplicates, sorting and indexing was carried out with *samtools* version 0.1.19 (http://samtools.sourceforge.net/). Alignments were visualised using the IGV browser version 2.3.12 (http://www.broadinstitute.org/igv/).

The mean read number was 5.8 M reads, with a s.d. of 1.6 M reads. Only 3% of the sequenced samples had less than 4.2 M reads (mean–s.d.), 11% of the samples had more than 7.3 M reads (mean+s.d.). The mean alignment rate was 87%, while the mean unique alignment rate was 75%, indicating that most of the reads could be mapped specifically (see Data Citation 1 for.bam files). The exact values, along with values for the different subsets, can be found in Table 1.

### Specificity and sensitivity

In total, the captured reads aligned to 1184 distinct regions of the *B. napus* ‘Darmor-Bzh’ v4.1 reference genome with a mean coverage of >10. Of these, a total of 637 regions were annotated as genes, and 184 corresponded to the intended target genes. Two target gene copies (*Bna.VIN3.A01* and *Bna.VIN3.C01*) had insufficient coverage, but were nevertheless included in the target list. A total of 33 regions were identified as targets giving a BLAST hit to the *FT* promotor. A further 12 regions were identified as pseudogenes of the target genes by blasting the gene sequences to the genome. Therefore, the target included 231 regions. The average target sensitivity^17^, interpreted as the percentage of target bases covered by sequence reads, was 85.6%. The average target specifity^17^, or the percentage of bases mapping to the intended target, was 68.0%, and therewith indeed better than in the foregoing experiment^16^. The target regions had a mean coverage of 670x and a mean normalized coverage of 533x. The mean target coverage over the mean genome-wide coverage, also called the enrichment factor, was about 1206, and therefore also increased compared to the preliminary experiment^16^ (for an overview of values see Table 2).

### Detection of SNPs and InDels

Calling of single nucleotide polymorphisms (SNPs) was performed with the algorithm mpileup in the *samtools* toolkit. SNPs were filtered for a minimum mapping quality of 50 and a read depth of ≥10, using vcftools (https://vcftools.github.io/man_latest.html).

For InDel calling, a separate mapping was performed using Bowtie2 (http://sourceforge.net/projects/bowtie-bio/files/bowtie2/), as described in ref. 22. Removal of duplicates, sorting and indexing was carried out with *samtools* version 0.1.19 (http://samtools.sourceforge.net/). An initial InDel calling was performed using *samtools* mpileup, and realignment of reads around InDels was performed using GATK (https://www.broadinstitute.org/gatk/). A final InDel calling was then performed as described above. InDels were filtered for a minimum mapping quality of 30 and a read depth of 10 or more using vcftools (https://vcftools.github.io/man_latest.html). SNP and InDel annotation was performed using CooVar^23^.

**Figure 1:**
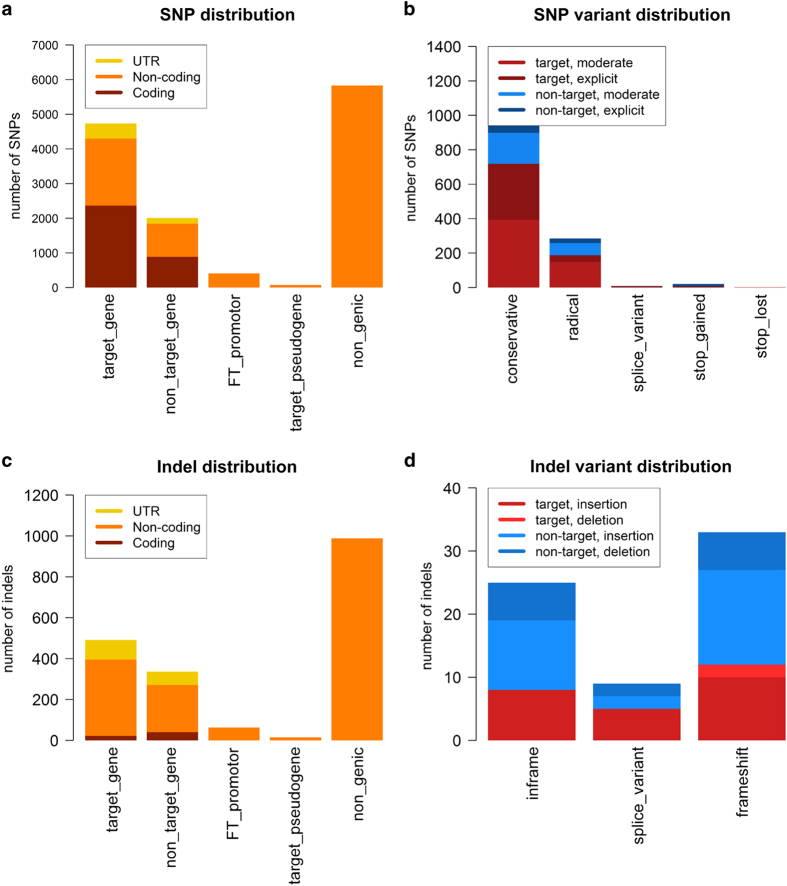
SNP and InDel distribution for all called SNPs and InDels meeting minimum quality requirements in the 1184 regions captured by deep sequencing. (**a**,**c**) Distribution to all analyzed regions and respective genic regions for SNPs (**a**) and InDels (**c**). (**b**,**d**): Distribution of nonsynonymous SNPs and InDels to different annotation classes regions for SNPs (**b**) and InDels (**d**).

We called and annotated 13053 SNPs among the 1184 captured regions, of which 5,216 were located in the target regions. Of those, 56 SNPs were either radical mutations, splice variants or stop codon mutations (gain or loss). InDel calling revealed a total of 1894 InDels, with 569 in the target regions. Only 25 InDels were frameshifts, amino acid insertions or splice variants. Potential functional variation was revealed in all homolog groups, although 7 homologs were completely conserved, while 16 carried only silent or synonymous variation. The distribution of variants on target and non-target regions is shown in Figure 1.

### CNV detection

Enriched regions and coverage differences were calculated using the *bedtools* software with multiBamCov (http://bedtools.readthedocs.org/en/latest/). Read coverage for each enriched region was normalised as follows:

coveragenorm=(number of aligned reads per region*total length of genome)/(total number of aligned reads*region length).

Copy number variation (CNV) in a given region was assumed if the ratio of normalised coverage(genotype)/mean normalised coverage (all genotypes) was smaller than 0.5 or higher than 1.5, respectively.

**Figure 2:**
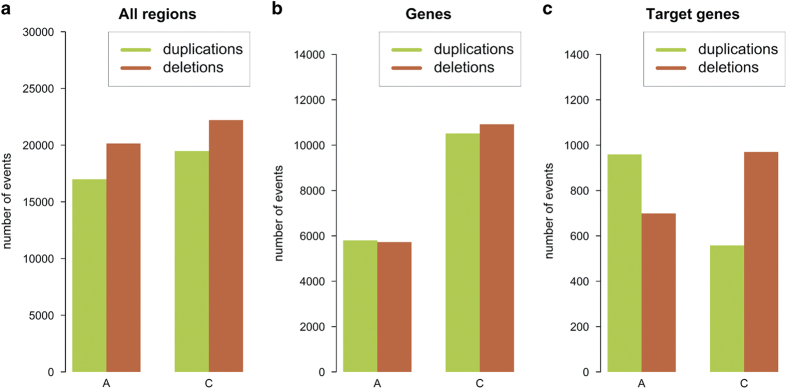
Distribution of deletions and duplications across the A and C subgenomes, respectively, among the 280 genotypes of the *B. napus* sequence capture diversity panel for (**a**) all captured regions, (**b**) all captured genes, and (**c**) all target genes.

No homolog group was found without CNV, and only two accessions were found which did not carry any CNV among the homologous copies of the target genes. Whereas it is therefore very unlikely to find two genotypes with exactly the same copy numbers, the frequency of a specific CNV among the population is generally low; about 87% of the CNVs had an abundance across the whole population of <10%. Deletions were generally more abundant than duplications (Figure 2) both for the A and C subgenomes, regarding all regions. This is expected, as genomes are known to reduce their gene space after polyploidization^24^, and corresponds to similar findings from whole-genome resequencing in a diverse panel of synthetic and natural *B. napus* (Samans *et al.*, submitted). Regarding only genes, duplication and deletion events were almost twice as frequent among C subgenome than A subgenome homologs. This appears surprising, as although the C subgenome is significantly larger^25,26^, its gene content is comparable to that of the A subgenome. A possible explanation for this apparent genome bias might be that transposons are more active in the C subgenome^26^, increasing the frequency of small-scale homology and double-strand breaks. Considering only the target genes from the flowering-time network, the ratio was strikingly different, with more duplications of A-subgenome genes than C-subgenome genes, but more deletions of C-subgenome genes than A-subgenome target genes. This indicates that A-subgenome copies had been selected over C-subgenome copies, while the other direction is less frequent. A corresponding bias was also observed by Samans *et al.* (submitted), who suggested that the size difference between homeologous segments from the A and C subgenomes is the driving force for the directional bias, with HNRT tending to replace larger C-subgenome segments by their smaller homoeologous segments from the A-subgenome. Another possible cause could be that gene copies which co-evolved in the progenitor genomes interact more effectively with each other than they do with copies from the other progenitor, a mechanism already assumed by other authors^27^. C-subgenome copies might have been affected more often due to the high transposon content, invoking a selective advantage for homeologous exchanges that replaced C-subgenome homologs with A-subgenome copies. As flowering is a major lifecycle axis, flowering genes are under strong selection and should show such an effect more clearly than random genes.

**Figure 3:**
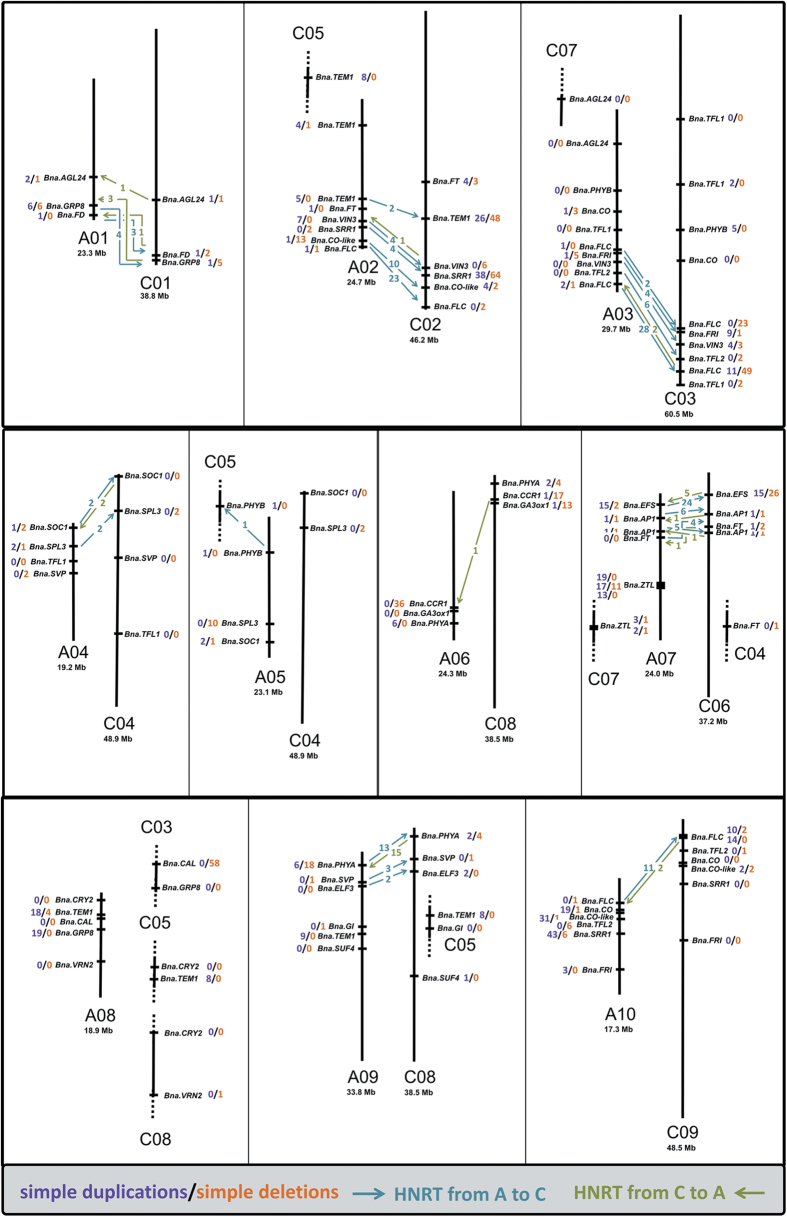
Landscape of homeologous target gene copies (excluding copies on x_random chromosomes). Chromosomes are shown as black vertical lines. Partial chromosomes are indicated by dotted lines at their ends. For chromosomes shown in full length, the total length is given below the chromosome name. Genetic positions are relative approximations. Arrows indicate HNRTs between homeologous copies, either from A-subgenome to C-subgenome homeologs (blue) or from C-subgenome to A-subgenome homeologs (green). The numbers behind the gene names indicate the number of simple duplication or deletion events (not involved in HNRTs) in purple and orange, respectively.

Homeologous gene replacements associated with putative HNRTs were observed frequently among the dataset. Excluding gene copies on non-assembled reference chromosomes (‘x_random’), we found 201 putative HNRT events between homeologous target gene copies, including 165 from A-subgenome to C-subgenome homeologs and 36 from C-subgenome to A-subgenome homeologs. In contrast, we found 448 simple duplications for the same gene copies (259 on A-subgenome and 189 on C-subgenome homeologs) and 490 simple deletions (139 on A-subgenome and 351 on C-subgenome homeologs). Although there is a low but significant correlation (r=0.68***) between the number of simple deletions and duplications on the C subgenome, such a correlation was not observed for the A subgenome. The highest rate of HNRTs was found between the strongly homeologous chromosomes A02/C02 and A03/C03, a finding in line with other authors^1^. On the other side of the spectrum, gene copies on chromosome A08 could not be associated with HNRTs to any homeologous copies, and only one putative HNRT each was observed for chromosomes A05 and A06, respectively. High HNRT frequencies were only observed at the very ends of chromosomes, whereas gene copies located closer to the center of the chromosomes were generally not involved in exchanges (Figure 3). Similar patterns have been frequently observed in *Brassicaceae*^1,28^.

### Code availability

All custom codes are supplied at https://github.com/ ‘targeted-deep-sequencing’.

## Data Records

The aligned sequence data (.bam files) and metadata are stored at the NCBI Sequence Read Archive database, SRP-Study accession SRP087610 (Data Citation 1).

## Technical Validation

All biallelic SNP, InDel and CNV data were recoded and included in a population structure analysis together with data from the Brassica 60 K Illumina Infinium SNP array (Clarke *et al.* 2016, accepted, Mason *et al.* 2016, accepted). Altogether these include 28,698 markers from the 60k SNP array and 12776 SNPs, 1894 InDels and 366 CNV markers from the present deep sequencing dataset, making a total of 43,733 markers. After pre-processing to filter for non-missing marker values >0.9, minor allele frequency >0.01 and non-missing individual markers >0.8, a total of 33,944 markers and 271 individuals were left, with 30.1% of the markers coming from the deep sequencing data. The subsequent principal component analysis for population structure revealed three main clusters. One cluster contained 137 winter-type *B. napus* accessions, one contained 93 spring-types and one semi-winter type accession, and the last cluster contained 40 genotypes of mixed origin, comprising mostly beet-forming and semi-winter types. We conclude i) that the variants called from the present deep sequencing dataset represent the species-wide diversity present in *B. napus*, in accordance with genotyping data from the SNP genotyping array, and ii) that the data correctly describe the study population.

In cooperation with Eleri Tudor from the John Innes Centre, Norwich, the data were compared to three Sanger-sequenced copies of *Bna.FRI* for 13 (A03 copy), 10 (A10 copy) and 9 (C03 copy) of our genotypes (unpublished data). The overall concordance rate between the data sets was 86%. 5 SNPs on the A03 copy had extremely low concordance rates, possibly due to a mapping problem. Removing those lead to an overall concordance rate of 90%.

We also checked for co-localizing SNPs between the datasets from the SNP genotyping array and from the deep sequencing. A total of 48 suitable SNP pairs were identified which showed apparently corresponding chromosome positions according to the BLAST search. Among this set, 3 SNP pairs had absolutely no correlation, presumably due to incorrect BLAST positions of the array SNP. For the remaining 45 SNPs, we observed a mean concordance of 91.3%, ranging from 83.0 to 97.7% with a standard deviation of 3.5%. These minor discrepancies may arise from the use of different selfing generations of some individuals for the array genotyping and the deep-sequencing. On the other hand, we noticed that concordance was lowest for SNPs discriminating between C and T, potentially suggesting a differential methylation of cytosine nucleotides.

## Usage Notes

The plant material described in this paper is publically available and can be made accessible upon request.

## Additional Information

**How to cite this article**: Schiessl, S. *et al.* Targeted deep sequencing of flowering regulators in *Brassica napus* reveals extensive copy number variation. *Sci. Data* 4:170013 doi: 10.1038/sdata.2017.13 (2017).

**Publisher’s note**: Springer Nature remains neutral with regard to jurisdictional claims in published maps and institutional affiliations.

## Supplementary Material



Supplementary Information

## Figures and Tables

**Table 1:** Sequencing and alignment values for total read number, alignment rate and unique alignment rate.

	**total reads**	**alignment rate [%]**	**unique alignment rate [%]**
**mean**	5768702	87.00	75.11
*winter*	5346938	89.21	77.04
*semi*	7023642	82.56	71.30
*spring*	6096897	84.82	73.25
*swede*	7038926	83.84	71.85
**min**	3681313	70.50	58.97
*winter*	4133157	74.85	63.33
*semi*	4298795	70.50	61.49
*spring*	3681313	72.18	58.97
*swede*	4691991	81.73	69.25
			
**max**	13869129	92.51	81.52
*winter*	9010533	92.51	81.52
*semi*	13839526	88.23	76.42
*spring*	13869129	91.37	81.37
*swede*	13724355	87.69	75.37
			
**std dev**	1581627	3.38	3.48
*winter*	910522	2.03	2.55
*semi*	3153296	5.16	4.28
*spring*	1807938	2.72	3.08
*swede*	2355237	1.73	1.85
The table lists the arithmetic mean, the minimal and maximal values as well as the s.d. for the total set (first line) and the respective subsets of winter type rapeseed, semi-winter rapeseed, spring type rapeseed and swedes.			

**Table 2:** Quality measures for the target enrichment for the total population and its subsets.

	**Total**	**winter**	**semi**	**spring**	**swede**
Mean genome-wide coverage	0.6	0.5	0.6	0.6	0.7
Mean target coverage	668.3	641.5	754.7	688.0	756.3
Enrichment factor	1205.9	1212.7	1199.0	1202.2	1158.9
Normalized mean target coverage	532.7	541.6	522.4	524.8	507.0
Fraction of target covered (%)	85.6	86.2	84.9	84.9	85.0
Reads covering target (%)	68.0	69.2	66.4	67.2	64.8
Genome fraction covered by >10 reads (%)	0.2	0.2	0.2	0.2	0.3
Fraction of target covered by > 10 reads (%)	79.0	79.8	78.0	78.1	78.7
The table lists mean values for genome-wide and target coverage, the enrichment factor calculated from that, the normalized mean target coverage, the fractions of the genome and the target covered by any read and by at least 10 reads.					
